# Groove pancreatitis mimicking duodenal malignancy with gastric outlet obstruction: successful conservative management to avoid unnecessary pancreaticoduodenectomy

**DOI:** 10.1093/jscr/rjag420

**Published:** 2026-06-03

**Authors:** Tushar Saini, Kumar Kaushik, Isha Shah

**Affiliations:** Department of General Surgery (GI Surgery Unit), Rohilkhand Medical College and Hospital, Pilibhit Bypass Road (Pawan Vihar / Mahanagar area), Bareilly District, Bareilly 243006, Uttar Pradesh, India; Department of General Surgery, Rohilkhand Medical College and Hospital, Pilibhit Bypass Road (Pawan Vihar / Mahanagar area), Bareilly District, Bareilly 243006, Uttar Pradesh, India; Department of General Surgery, Rohilkhand Medical College and Hospital, Pilibhit Bypass Road (Pawan Vihar / Mahanagar area), Bareilly District, Bareilly 243006, Uttar Pradesh, India

**Keywords:** groove pancreatitis, gastric outlet obstruction, conservative management, pancreaticoduodenectomy

## Abstract

Groove pancreatitis (GP) is a rare focal form of chronic pancreatitis that frequently mimics pancreatic head or duodenal malignancy, often leading to unnecessary pancreaticoduodenectomy. We report the case of a 47-year-old man with heavy alcohol use who presented with severe gastric outlet obstruction (GOO) due to a mass-like duodenal lesion. Clinical, endoscopic, and radiologic features initially suggested primary duodenal malignancy. However, characteristic imaging, negative biopsies, and multidisciplinary review confirmed segmental GP. The patient achieved complete resolution of GOO and the duodenal lesion within 7 days using conservative management alone (alcohol cessation, nasogastric decompression, proton-pump inhibitors, pancreatic enzymes, and nutritional support). This unusually rapid and morbidity-free response highlights that, when malignancy is reliably excluded, pragmatic conservative therapy can safely prevent major pancreatic resection and its associated costs. Greater awareness of GP and early multimodal assessment can reduce overtreatment in ambiguous pancreaticoduodenal masses.

## Introduction

**Figure 1:**
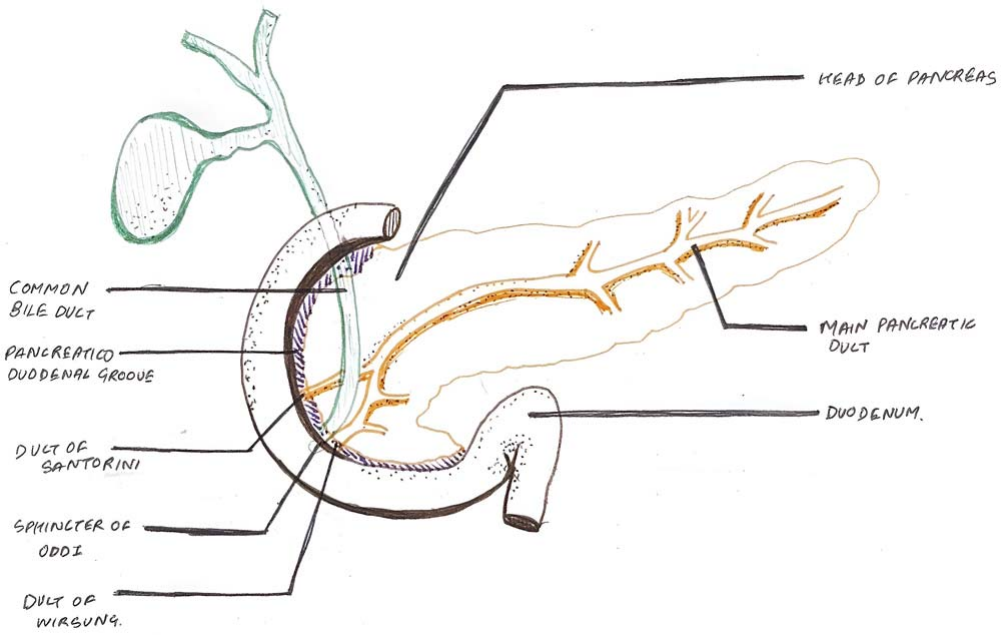
Illustrative diagram showing the pancreaticoduodenal groove, the potential space between the medial wall of the duodenum and the pancreatic head.

Chronic pancreatitis is a progressive fibroinflammatory disorder characterized by irreversible parenchymal damage, exocrine insufficiency, and eventual endocrine dysfunction. Groove pancreatitis (GP), also known as paraduodenal pancreatitis [1], is a rare focal variant confined to the anatomic groove between the pancreatic head, duodenum, and common bile duct (Fig. 1).

Originally described in the German literature as ‘Rinnen-pankreatitis’, the term ‘groove pancreatitis’ was coined by Stolte *et al*. in 1982 [2, 3]. GP is classified into pure and segmental forms. The pure form is limited to the groove with sparing of the pancreatic head parenchyma, whereas the segmental form involves both the groove and the medial aspect of the pancreatic head. A diffuse variant with prominent groove involvement in the setting of generalized chronic pancreatitis has also been described [4]. Histopathologically, GP may present as cystic, solid (mass-forming), or mixed types [5]. Characteristic contrast-enhanced computed tomography (CECT) findings include sheet-like hypoenhancement in the pancreaticoduodenal groove and circumferential duodenal wall thickening; Magnetic resonance imaging (MRI) shows T_2_-hyperintense cysts [6].

GP classically mimics pancreatic head or periampullary carcinoma clinically and radiologically, frequently resulting in unnecessary pancreaticoduodenectomy despite benign pathology [7, 8]. Although conservative management achieves symptom resolution in 35%–50% of confirmed cases, surgery remains overutilized when preoperative certainty is lacking [9, 10]. This case report describes an unusual presentation of segmental GP manifesting as near-complete gastric outlet obstruction (GOO) that closely mimicked primary duodenal malignancy. It demonstrates rapid, complete resolution with conservative therapy, thereby avoiding the well-documented morbidity and healthcare costs of major resection [11] and addressing a critical gap in surgical decision-making.

## Case presentation

A 47-year-old man with a 10-year history of heavy alcohol consumption (50–60 ml ethanol daily) presented with 1-month progressive epigastric pain and intractable vomiting (10–12 episodes/day for 5 days) containing coffee-ground material and undigested food, accompanied by significant weight loss. Examination revealed epigastric fullness and positive succussion splash. Nasogastric aspiration yielded 800 ml of dark-brown fluid, confirming GOO with upper gastrointestinal bleeding.

**Figure 2:**
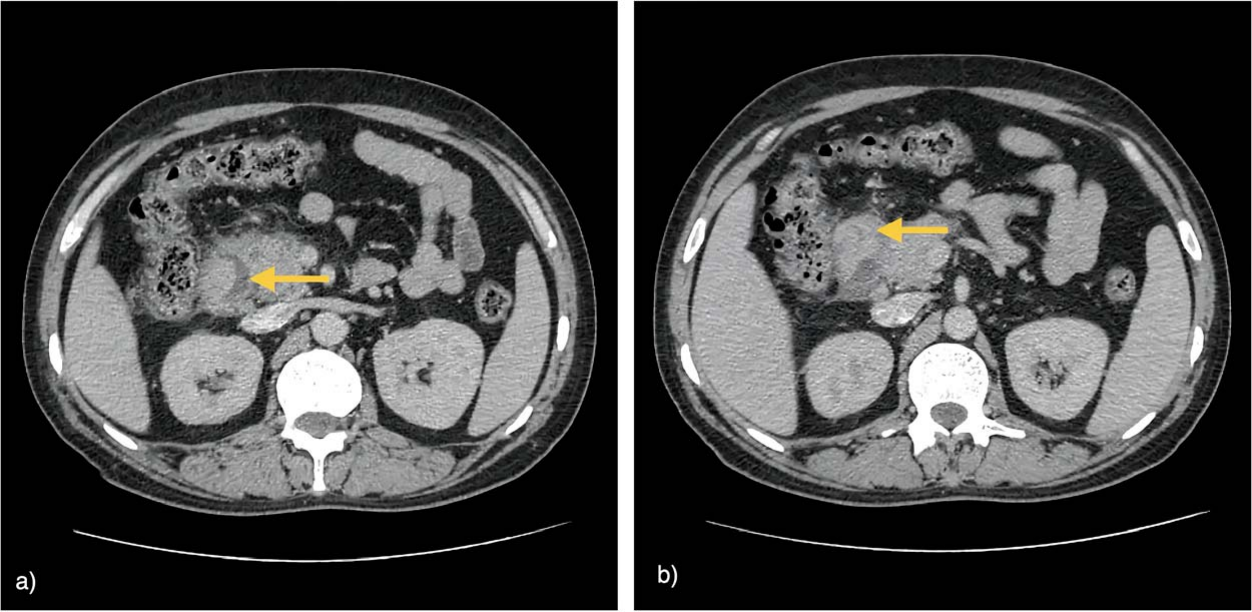
CECT of the abdomen. (a) Axial image showing sheet-like hypoenhancement (arrow) in the pancreaticoduodenal groove with a bulky pancreatic head. (b) Axial image demonstrating irregular, heterogeneously enhancing circumferential duodenal wall thickening at the D1–D2 junction (arrow) causing luminal narrowing with surrounding fat stranding.

**Figure 3:**
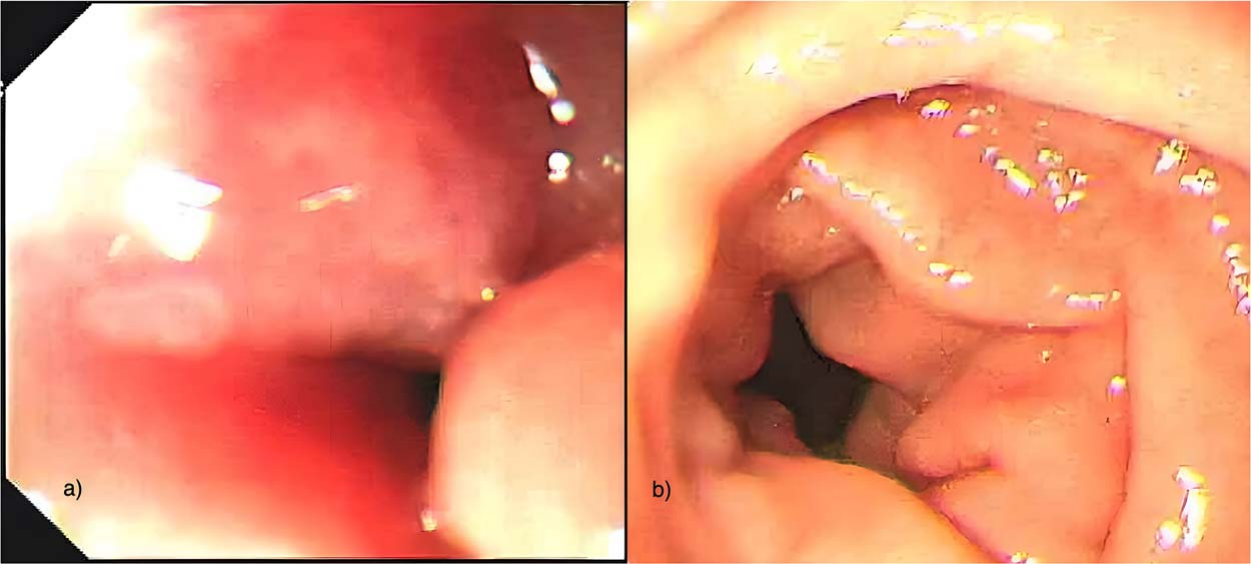
Upper gastrointestinal endoscopy. (a) Mass-like circumferential thickening and narrowing at the junction of the first and second parts of the duodenum (D1–D2) causing near-complete luminal obstruction. (b) Follow-up endoscopy 1 week after conservative management showing substantial regression of the duodenal lesion with complete restoration of luminal patency.

Serum amylase was transiently elevated (347 IU/l) but normalized within 1 week. CECT demonstrated sheet-like hypoenhancement in the pancreaticoduodenal groove, bulky pancreatic head, and circumferential duodenal wall thickening (3.3 × 1.5 × 4.4 cm) with fat stranding (Fig. 2), initially interpreted as favouring neoplasm. Upper gastrointestinal endoscopy showed a mass-like duodenal lesion at the D1–D2 junction causing near-complete obstruction (Fig. 3a); biopsies revealed only chronic nonspecific duodenitis.

Multidisciplinary tumour-board review of clinical, endoscopic, radiologic, and histologic data confirmed segmental groove pancreatitis. The patient was managed conservatively with total alcohol abstinence, nasogastric decompression, intravenous fluids, analgesics, proton-pump inhibitors, pancreatic enzyme supplementation, and nutritional support. Dramatic improvement occurred within 7 days: nasogastric output decreased, oral intake resumed, and bowel function normalized. Repeat endoscopy confirmed complete resolution of stenosis (Fig. 3b). Endoscopic ultrasound showed reduced duodenal wall thickness, and repeat biopsies remained benign. Using the M-ANNHEIM severity index [12], the patient improved from stage B (10 points) to stage A (3 points) by Day 15, with documented weight gain.

This nonoperative pathway was deliberately chosen to avert the morbidity and costs of pancreaticoduodenectomy, which is frequently performed for presumed malignancy in similar presentations. No surgery was required, and the patient remained symptom-free on follow-up.

## Discussion

The diagnosis of segmental groove pancreatitis was established by integrating heavy alcohol history, transient amylase elevation, characteristic imaging and endoscopic findings, repeatedly negative biopsies, and—most convincingly—rapid clinical and endoscopic resolution with conservative management alone.

This case illustrates the diagnostic challenge of GP when segmental disease produces a mass-like duodenal lesion causing severe GOO—a rare but recognized complication [13]. Beyond its common resemblance to pancreatic head adenocarcinoma, the initial presentation of GP in this case closely simulated primary duodenal malignancy [7]. Such pseudotumoral presentations frequently drive unnecessary operative intervention [8].

Chronic heavy alcohol use promotes fibroinflammatory scarring within the groove [4]. The patient’s weight loss, coffee-ground vomiting, and high-volume nasogastric aspirate reflected severe GOO secondary to inflammatory duodenal stenosis. Consistent with systematic evidence supporting a stepwise approach, conservative management was initiated as the initial strategy in this patient with confirmed groove pancreatitis after malignancy was excluded [10]. In this case, the chosen regimen produced complete resolution of stenosis within 7 days, reduction in duodenal wall thickness on endoscopic ultrasound, and M-ANNHEIM improvement, confirming the benign aetiology and rendering surgery unnecessary.

Although conservative therapy achieves symptom resolution in 35%–50% of cases, systematic reviews show that 50%–60% of patients ultimately undergo pancreaticoduodenectomy despite benign pathology [9, 10]. These resections carry 20%–40% morbidity, long-term exocrine/endocrine insufficiency, prolonged hospital stays, and high costs [11]. When preoperative certainty is achieved, pragmatic conservative management offers comparable outcomes with minimal risk and negligible cost in responsive patients [14].

GP rarely coexists with malignancy [15]. In the vast majority of patients with reliably excluded cancer, early conservative therapy safely prevents unnecessary major resection. What distinguishes this report is the documentation of an unusually rapid (<7 days) and complete nonoperative resolution in a GOO presentation that closely mimicked primary duodenal malignancy. It supports a paradigm shift towards multimodal assessment (detailed history, high-quality imaging, biopsy, multidisciplinary review, and short-interval re-evaluation) in ambiguous pancreaticoduodenal masses, thereby reducing patient morbidity, improving quality of life, and optimizing resource allocation.
